# Crystal structure of pencycuron

**DOI:** 10.1107/S2056989015012414

**Published:** 2015-07-04

**Authors:** Gihaeng Kang, Jineun Kim, Eunjin Kwon, Tae Ho Kim

**Affiliations:** a Department of Chemistry and Research Institute of Natural Sciences, Gyeongsang, National University, Jinju 660-701, Republic of Korea

**Keywords:** crystal structure, pencycuron, urea, fungicide, hydrogen bonding, π–π inter­actions

## Abstract

In the title compound [systematic name: 1-(4-chloro­benz­yl)-1-cyclo­pentyl-3-phenyl­urea], C_19_H_21_ClN_2_O, which is a urea fungicide, the cyclo­pentyl ring adopts an envelope conformation, with one of the methyl­ene C atoms adjacent to the C atom bonding to the N atom as the flap. The dihedral angles between the mean planes of the central cyclo­pentyl ring (all atoms) and the chloro­benzyl and phenyl rings are 77.96 (6) and 57.77 (7)°, respectively. In the crystal, N—H⋯O hydrogen bonds link adjacent mol­ecules, forming *C*(4) chains propagating along the *b*-axis direction. The chains are linked by weak π–π inter­actions between the chloro­benzene rings [centroid–centroid separation = 3.9942 (9) Å], resulting in two-dimensional networks extending parellel to the (110) plane.

## Related literature   

For information on the fungicidal properties of the title compound, see: Pal *et al.* (2005 ▸). For a related crystal structure, see: Bjerglund *et al.* (2012 ▸).
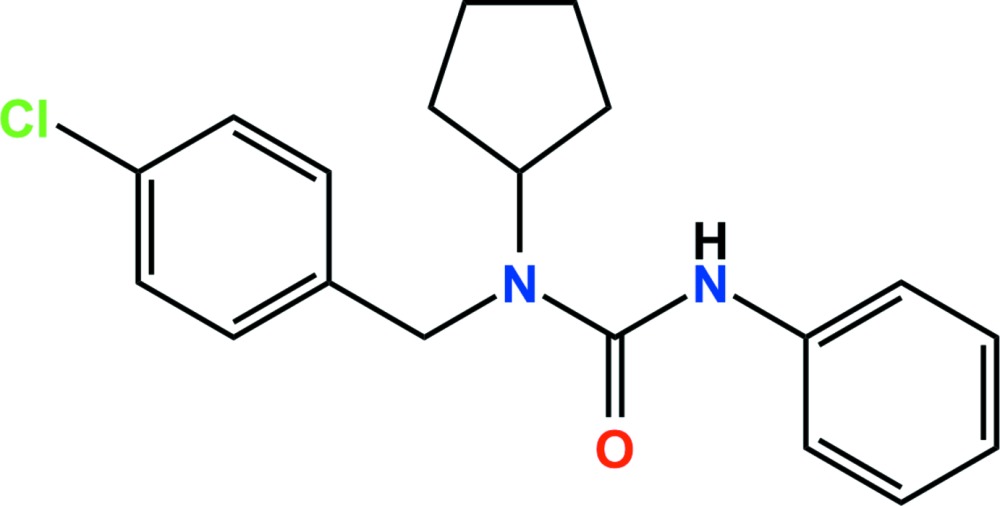


## Experimental   

### Crystal data  

C_19_H_21_ClN_2_O
*M* *_r_* = 328.83
Orthorhombic, 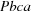

*a* = 12.1585 (5) Å
*b* = 8.6721 (4) Å
*c* = 32.6152 (12) Å
*V* = 3438.9 (2) Å^3^
*Z* = 8
Mo *K*α radiation
μ = 0.23 mm^−1^
*T* = 173 K
0.50 × 0.11 × 0.09 mm

### Data collection  

Bruker APEX-II CCD diffractometer
Absorption correction: multi-scan (*SADABS*; Bruker, 2009 ▸) *T*_min_ = 0.894, *T*_max_ = 0.980
27037 measured reflections
3374 independent reflections
2698 reflections with *I* > 2σ(*I*)
*R*_int_ = 0.039

### Refinement  

*R*[*F* ^2^ > 2σ(*F* ^2^)] = 0.037
*wR*(*F* ^2^) = 0.095
*S* = 1.04
3374 reflections
212 parameters
H atoms treated by a mixture of independent and constrained refinement
Δρ_max_ = 0.17 e Å^−3^
Δρ_min_ = −0.24 e Å^−3^

### 

Data collection: *APEX2* (Bruker, 2009 ▸); cell refinement: *SAINT* (Bruker, 2009 ▸); data reduction: *SAINT*; program(s) used to solve structure: *SHELXS97* (Sheldrick 2008 ▸); program(s) used to refine structure: *SHELXL2013* (Sheldrick, 2015 ▸); molecular graphics: *DIAMOND* (Brandenburg, 2010 ▸); software used to prepare material for publication: *SHELXTL* (Sheldrick 2008 ▸).

## Supplementary Material

Crystal structure: contains datablock(s) global, I. DOI: 10.1107/S2056989015012414/hb7456sup1.cif

Structure factors: contains datablock(s) I. DOI: 10.1107/S2056989015012414/hb7456Isup2.hkl

Supporting information file. DOI: 10.1107/S2056989015012414/hb7456Isup3.cml

**Fig. 1.**
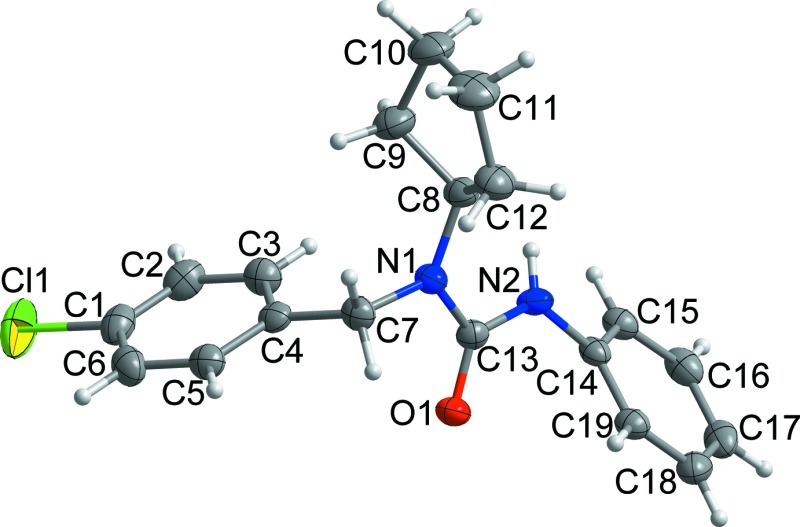
The asymmetric unit of the title compound with displacement ellipsoids drawn at the 50% probability level.

**Fig. 2.**
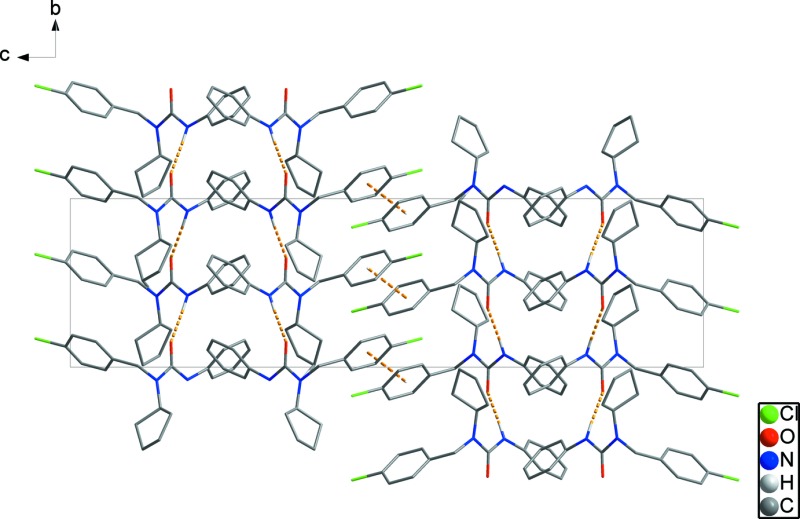
Crystal packing viewed along the a axis. The intermolecular N—H···O hydrogen bonds and weak π–π interactions are shown as dashed lines.

. DOI: 10.1107/S2056989015012414/hb7456fig1.tif
The asymmetric unit of the title compound with displacement ellipsoids drawn at the 50% probability level.

a . DOI: 10.1107/S2056989015012414/hb7456fig2.tif
Crystal packing viewed along the *a* axis. The inter­molecular N—H⋯O hydrogen bonds and weak π–π inter­actions are shown as dashed lines.

CCDC reference: 1409194

Additional supporting information:  crystallographic information; 3D view; checkCIF report

## Figures and Tables

**Table 1:** Hydrogen-bond geometry (, )

*D*H*A*	*D*H	H*A*	*D* *A*	*D*H*A*
N2H2*N*O1^i^	0.828(19)	2.081(19)	2.8838(17)	163.1(17)

Symmetry code: (i) 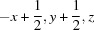.

## supplementary crystallographic information

### S1. Comment

Pencycuron, [systematic name: 1-(4-chlorobenzyl)-1-cyclopentyl-3-phenylurea], is a urea fungicide and it has been used for the control of diseases in various crops, including rice (Pal *et al.*, 2005). However, until now its crystal structure has not been reported. In the title compound (Fig. 1), the dihedral angles between the mean planes of the central cyclopentyl ring [r.m.s. deviation = 0.1693] and the chlorobenzyl and phenyl rings are 77.96 (6) and 57.778 (7)°, respectively. All bond lengths and bond angles are normal and comparable to those observed in the crystal structure of a similar compound (Bjerglund *et al.*, 2012).

In the crystal (Fig. 2), N—H···O hydrogen bonds link adjacent molecules, forming a one-dimensional chains along the *b* axis direction (Table. 1). The chains are linked by weak π–π interactions [*Cg*1···*Cg*1^ii^, 3.9942 (9) Å], resulting in a two-dimensional networks parellel to the (110) plane (*Cg*1 is the centroid of the C1–C6 ring)[for symmetry codes: (ii), -*x*, -*y*, -*z*].

### S2. Experimental

The title compound was purchased from the Dr Ehrenstorfer GmbH Company. Slow evaporation of a solution in CH_2_Cl_2_ gave colourless needles.

### S3. Refinement

The N-bound H atom was located in a difference Fourier map and freely refined (N—H = 0.828 (19) Å). The C-bound H atoms were positioned geometrically and refined using a riding model with d(C—H) = 1.00 Å, *U*_iso_ = 1.2*U*_eq_(C) for C*sp*^3^—H, d(C—H) = 0.99 Å, *U*_iso_ = 1.2*U*_eq_(C) for CH_2_ groups, d(C—H) = 0.95 Å, *U*_iso_ = 1.2*U*_eq_(C) for aromatic C—H.

### Crystal data

**Table:**

C_19_H_21_ClN_2_O	*D*_x_ = 1.270 Mg m^−^^3^
*M**_r_* = 328.83	Mo *K*α radiation, λ = 0.71073 Å
Orthorhombic, *P**b**c**a*	Cell parameters from 6227 reflections
*a* = 12.1585 (5) Å	θ = 2.5–27.3°
*b* = 8.6721 (4) Å	µ = 0.23 mm^−^^1^
*c* = 32.6152 (12) Å	*T* = 173 K
*V* = 3438.9 (2) Å^3^	Needle, colourless
*Z* = 8	0.50 × 0.11 × 0.09 mm
*F*(000) = 1392	

### Data collection

**Table:**

Bruker APEX-II CCD diffractometer	2698 reflections with *I* > 2σ(*I*)
φ and ω scans	*R*_int_ = 0.039
Absorption correction: multi-scan (*SADABS*; Bruker, 2009)	θ_max_ = 26.0°, θ_min_ = 2.1°
*T*_min_ = 0.894, *T*_max_ = 0.980	*h* = −14→14
27037 measured reflections	*k* = −6→10
3374 independent reflections	*l* = −40→40

### Refinement

**Table:**

Refinement on *F*^2^	0 restraints
Least-squares matrix: full	Hydrogen site location: mixed
*R*[*F*^2^ > 2σ(*F*^2^)] = 0.037	H atoms treated by a mixture of independent and constrained refinement
*wR*(*F*^2^) = 0.095	*w* = 1/[σ^2^(*F*_o_^2^) + (0.0384*P*)^2^ + 1.2518*P*] where *P* = (*F*_o_^2^ + 2*F*_c_^2^)/3
*S* = 1.04	(Δ/σ)_max_ < 0.001
3374 reflections	Δρ_max_ = 0.17 e Å^−^^3^
212 parameters	Δρ_min_ = −0.24 e Å^−^^3^

### Special details

**Table:**

Geometry. All e.s.d.'s (except the e.s.d. in the dihedral angle between two l.s. planes) are estimated using the full covariance matrix. The cell e.s.d.'s are taken into account individually in the estimation of e.s.d.'s in distances, angles and torsion angles; correlations between e.s.d.'s in cell parameters are only used when they are defined by crystal symmetry. An approximate (isotropic) treatment of cell e.s.d.'s is used for estimating e.s.d.'s involving l.s. planes.

### Fractional atomic coordinates and isotropic or equivalent isotropic displacement parameters (Å^2^)

**Table:**

	*x*	*y*	*z*	*U*_iso_*/*U*_eq_	
Cl1	0.23590 (4)	−0.16966 (8)	−0.05434 (2)	0.06131 (19)	
O1	0.15878 (8)	−0.14846 (11)	0.15909 (3)	0.0287 (2)	
N1	0.08966 (10)	0.07872 (15)	0.13656 (4)	0.0278 (3)	
N2	0.23017 (10)	0.07453 (16)	0.18515 (4)	0.0288 (3)	
H2N	0.2489 (14)	0.162 (2)	0.1777 (5)	0.039 (5)*	
C1	0.17668 (14)	−0.1232 (2)	−0.00710 (5)	0.0384 (4)	
C2	0.22919 (13)	−0.0213 (2)	0.01841 (5)	0.0397 (4)	
H2	0.2975	0.0233	0.0106	0.048*	
C3	0.18127 (12)	0.0160 (2)	0.05576 (5)	0.0355 (4)	
H3	0.2173	0.0863	0.0736	0.043*	
C4	0.08150 (12)	−0.04807 (17)	0.06739 (4)	0.0277 (3)	
C5	0.03091 (13)	−0.15110 (19)	0.04097 (5)	0.0327 (4)	
H5	−0.0373	−0.1962	0.0486	0.039*	
C6	0.07769 (14)	−0.1895 (2)	0.00376 (5)	0.0390 (4)	
H6	0.0423	−0.2604	−0.0141	0.047*	
C7	0.02356 (12)	−0.00774 (19)	0.10708 (4)	0.0298 (3)	
H7A	−0.0428	0.0535	0.1005	0.036*	
H7B	−0.0012	−0.1045	0.1203	0.036*	
C8	0.06470 (12)	0.24352 (18)	0.14250 (5)	0.0315 (4)	
H8	0.1190	0.2854	0.1627	0.038*	
C9	0.07296 (15)	0.3403 (2)	0.10377 (5)	0.0436 (4)	
H9A	0.0381	0.2873	0.0802	0.052*	
H9B	0.1506	0.3629	0.0969	0.052*	
C10	0.01105 (17)	0.4869 (2)	0.11481 (6)	0.0532 (5)	
H10A	−0.0149	0.5406	0.0898	0.064*	
H10B	0.0584	0.5579	0.1307	0.064*	
C11	−0.08580 (17)	0.4315 (2)	0.14061 (7)	0.0565 (5)	
H11A	−0.1520	0.4182	0.1233	0.068*	
H11B	−0.1027	0.5070	0.1625	0.068*	
C12	−0.05145 (14)	0.2774 (2)	0.15928 (6)	0.0421 (4)	
H12A	−0.1033	0.1949	0.1511	0.050*	
H12B	−0.0502	0.2841	0.1896	0.050*	
C13	0.15966 (11)	−0.00577 (17)	0.16037 (4)	0.0249 (3)	
C14	0.29239 (11)	0.00718 (17)	0.21711 (4)	0.0255 (3)	
C15	0.39770 (12)	0.06323 (18)	0.22439 (5)	0.0300 (3)	
H15	0.4283	0.1387	0.2066	0.036*	
C16	0.45819 (13)	0.0096 (2)	0.25737 (5)	0.0357 (4)	
H16	0.5295	0.0500	0.2625	0.043*	
C17	0.41528 (14)	−0.1025 (2)	0.28280 (5)	0.0381 (4)	
H17	0.4569	−0.1396	0.3054	0.046*	
C18	0.31135 (14)	−0.16039 (19)	0.27514 (5)	0.0354 (4)	
H18	0.2822	−0.2390	0.2923	0.043*	
C19	0.24920 (12)	−0.10478 (18)	0.24270 (4)	0.0293 (3)	
H19	0.1772	−0.1434	0.2381	0.035*	

### Atomic displacement parameters (Å^2^)

**Table:**

	*U*^11^	*U*^22^	*U*^33^	*U*^12^	*U*^13^	*U*^23^
Cl1	0.0543 (3)	0.0950 (5)	0.0346 (2)	0.0261 (3)	0.0028 (2)	−0.0022 (3)
O1	0.0304 (5)	0.0214 (5)	0.0343 (6)	−0.0008 (5)	−0.0029 (4)	−0.0001 (5)
N1	0.0293 (6)	0.0238 (7)	0.0303 (6)	0.0018 (6)	−0.0084 (5)	−0.0012 (6)
N2	0.0295 (7)	0.0207 (6)	0.0361 (7)	−0.0033 (6)	−0.0097 (5)	0.0054 (6)
C1	0.0388 (9)	0.0465 (10)	0.0298 (8)	0.0157 (8)	−0.0022 (7)	0.0049 (8)
C2	0.0300 (8)	0.0476 (11)	0.0414 (9)	0.0029 (8)	0.0011 (7)	0.0082 (9)
C3	0.0303 (8)	0.0365 (9)	0.0396 (9)	−0.0018 (7)	−0.0046 (7)	−0.0005 (8)
C4	0.0274 (7)	0.0269 (8)	0.0290 (7)	0.0030 (7)	−0.0073 (6)	0.0041 (7)
C5	0.0300 (8)	0.0331 (9)	0.0350 (8)	−0.0003 (7)	−0.0070 (6)	0.0014 (7)
C6	0.0408 (9)	0.0412 (10)	0.0352 (8)	0.0044 (8)	−0.0113 (7)	−0.0056 (8)
C7	0.0254 (7)	0.0316 (9)	0.0324 (8)	−0.0024 (7)	−0.0056 (6)	−0.0017 (7)
C8	0.0354 (8)	0.0245 (8)	0.0345 (8)	0.0026 (7)	−0.0091 (7)	0.0022 (7)
C9	0.0495 (10)	0.0358 (10)	0.0454 (10)	0.0022 (8)	−0.0033 (8)	0.0120 (9)
C10	0.0705 (13)	0.0338 (10)	0.0552 (11)	0.0087 (10)	−0.0153 (10)	0.0118 (9)
C11	0.0532 (11)	0.0433 (12)	0.0730 (14)	0.0190 (10)	−0.0099 (10)	0.0020 (11)
C12	0.0459 (10)	0.0343 (9)	0.0459 (10)	0.0084 (8)	0.0025 (8)	−0.0006 (8)
C13	0.0231 (7)	0.0246 (8)	0.0269 (7)	−0.0011 (6)	0.0017 (6)	0.0016 (7)
C14	0.0265 (7)	0.0211 (7)	0.0290 (7)	0.0047 (6)	−0.0049 (6)	−0.0033 (6)
C15	0.0278 (8)	0.0253 (8)	0.0369 (8)	0.0015 (6)	−0.0026 (6)	−0.0027 (7)
C16	0.0271 (7)	0.0346 (9)	0.0454 (9)	0.0065 (7)	−0.0104 (7)	−0.0104 (8)
C17	0.0430 (9)	0.0375 (10)	0.0339 (8)	0.0145 (8)	−0.0141 (7)	−0.0038 (8)
C18	0.0440 (9)	0.0297 (9)	0.0326 (8)	0.0073 (8)	−0.0023 (7)	0.0018 (7)
C19	0.0286 (7)	0.0249 (8)	0.0342 (8)	0.0023 (7)	−0.0038 (6)	−0.0013 (7)

### Geometric parameters (Å, º)

**Table:**

Cl1—C1	1.7478 (17)	C8—H8	1.0000
O1—C13	1.2382 (17)	C9—C10	1.521 (3)
N1—C13	1.3654 (18)	C9—H9A	0.9900
N1—C7	1.4604 (18)	C9—H9B	0.9900
N1—C8	1.474 (2)	C10—C11	1.525 (3)
N2—C13	1.3686 (19)	C10—H10A	0.9900
N2—C14	1.4141 (18)	C10—H10B	0.9900
N2—H2N	0.828 (19)	C11—C12	1.527 (3)
C1—C2	1.371 (2)	C11—H11A	0.9900
C1—C6	1.380 (2)	C11—H11B	0.9900
C2—C3	1.389 (2)	C12—H12A	0.9900
C2—H2	0.9500	C12—H12B	0.9900
C3—C4	1.387 (2)	C14—C19	1.384 (2)
C3—H3	0.9500	C14—C15	1.390 (2)
C4—C5	1.385 (2)	C15—C16	1.384 (2)
C4—C7	1.515 (2)	C15—H15	0.9500
C5—C6	1.381 (2)	C16—C17	1.380 (2)
C5—H5	0.9500	C16—H16	0.9500
C6—H6	0.9500	C17—C18	1.382 (2)
C7—H7A	0.9900	C17—H17	0.9500
C7—H7B	0.9900	C18—C19	1.387 (2)
C8—C9	1.520 (2)	C18—H18	0.9500
C8—C12	1.543 (2)	C19—H19	0.9500
			
C13—N1—C7	116.23 (12)	H9A—C9—H9B	109.0
C13—N1—C8	125.02 (12)	C9—C10—C11	104.42 (15)
C7—N1—C8	118.11 (12)	C9—C10—H10A	110.9
C13—N2—C14	124.07 (13)	C11—C10—H10A	110.9
C13—N2—H2N	117.8 (12)	C9—C10—H10B	110.9
C14—N2—H2N	116.5 (12)	C11—C10—H10B	110.9
C2—C1—C6	121.24 (15)	H10A—C10—H10B	108.9
C2—C1—Cl1	119.44 (13)	C10—C11—C12	106.55 (15)
C6—C1—Cl1	119.32 (14)	C10—C11—H11A	110.4
C1—C2—C3	119.13 (15)	C12—C11—H11A	110.4
C1—C2—H2	120.4	C10—C11—H11B	110.4
C3—C2—H2	120.4	C12—C11—H11B	110.4
C4—C3—C2	120.91 (15)	H11A—C11—H11B	108.6
C4—C3—H3	119.5	C11—C12—C8	105.99 (14)
C2—C3—H3	119.5	C11—C12—H12A	110.5
C5—C4—C3	118.45 (14)	C8—C12—H12A	110.5
C5—C4—C7	118.30 (13)	C11—C12—H12B	110.5
C3—C4—C7	123.23 (14)	C8—C12—H12B	110.5
C6—C5—C4	121.28 (15)	H12A—C12—H12B	108.7
C6—C5—H5	119.4	O1—C13—N1	120.78 (13)
C4—C5—H5	119.4	O1—C13—N2	122.27 (13)
C1—C6—C5	118.98 (16)	N1—C13—N2	116.96 (13)
C1—C6—H6	120.5	C19—C14—C15	119.46 (13)
C5—C6—H6	120.5	C19—C14—N2	122.10 (13)
N1—C7—C4	115.17 (12)	C15—C14—N2	118.31 (14)
N1—C7—H7A	108.5	C16—C15—C14	120.32 (15)
C4—C7—H7A	108.5	C16—C15—H15	119.8
N1—C7—H7B	108.5	C14—C15—H15	119.8
C4—C7—H7B	108.5	C17—C16—C15	120.21 (15)
H7A—C7—H7B	107.5	C17—C16—H16	119.9
N1—C8—C9	114.36 (13)	C15—C16—H16	119.9
N1—C8—C12	114.81 (13)	C16—C17—C18	119.51 (15)
C9—C8—C12	104.49 (13)	C16—C17—H17	120.2
N1—C8—H8	107.6	C18—C17—H17	120.2
C9—C8—H8	107.6	C17—C18—C19	120.64 (16)
C12—C8—H8	107.6	C17—C18—H18	119.7
C8—C9—C10	103.43 (14)	C19—C18—H18	119.7
C8—C9—H9A	111.1	C14—C19—C18	119.83 (14)
C10—C9—H9A	111.1	C14—C19—H19	120.1
C8—C9—H9B	111.1	C18—C19—H19	120.1
C10—C9—H9B	111.1		
			
C6—C1—C2—C3	−0.2 (3)	C9—C10—C11—C12	24.7 (2)
Cl1—C1—C2—C3	179.54 (13)	C10—C11—C12—C8	−1.9 (2)
C1—C2—C3—C4	−0.2 (3)	N1—C8—C12—C11	−147.64 (15)
C2—C3—C4—C5	0.4 (2)	C9—C8—C12—C11	−21.56 (18)
C2—C3—C4—C7	−177.90 (15)	C7—N1—C13—O1	−6.1 (2)
C3—C4—C5—C6	−0.3 (2)	C8—N1—C13—O1	164.53 (14)
C7—C4—C5—C6	178.12 (14)	C7—N1—C13—N2	173.51 (12)
C2—C1—C6—C5	0.4 (3)	C8—N1—C13—N2	−15.8 (2)
Cl1—C1—C6—C5	−179.41 (12)	C14—N2—C13—O1	−13.1 (2)
C4—C5—C6—C1	−0.1 (2)	C14—N2—C13—N1	167.32 (13)
C13—N1—C7—C4	−81.80 (16)	C13—N2—C14—C19	−40.2 (2)
C8—N1—C7—C4	106.87 (15)	C13—N2—C14—C15	144.02 (15)
C5—C4—C7—N1	169.45 (13)	C19—C14—C15—C16	−1.1 (2)
C3—C4—C7—N1	−12.2 (2)	N2—C14—C15—C16	174.79 (14)
C13—N1—C8—C9	131.17 (15)	C14—C15—C16—C17	1.4 (2)
C7—N1—C8—C9	−58.34 (18)	C15—C16—C17—C18	−0.2 (2)
C13—N1—C8—C12	−108.04 (16)	C16—C17—C18—C19	−1.3 (2)
C7—N1—C8—C12	62.45 (18)	C15—C14—C19—C18	−0.3 (2)
N1—C8—C9—C10	163.14 (14)	N2—C14—C19—C18	−176.06 (14)
C12—C8—C9—C10	36.79 (18)	C17—C18—C19—C14	1.5 (2)
C8—C9—C10—C11	−38.08 (18)		

### Hydrogen-bond geometry (Å, º)

**Table:**

*D*—H···*A*	*D*—H	H···*A*	*D*···*A*	*D*—H···*A*
N2—H2*N*···O1^i^	0.828 (19)	2.081 (19)	2.8838 (17)	163.1 (17)

Symmetry code: (i) −*x*+1/2, *y*+1/2, *z*.
