# Supplementary figures and images for: Crystal structure of pencycuron
Source: Acta Crystallogr E Crystallogr Commun. 2015 Jul 4;71(Pt 8):o532. doi: 10.1107/S2056989015012414 (PMC4571381; doi:10.1107/S2056989015012414)

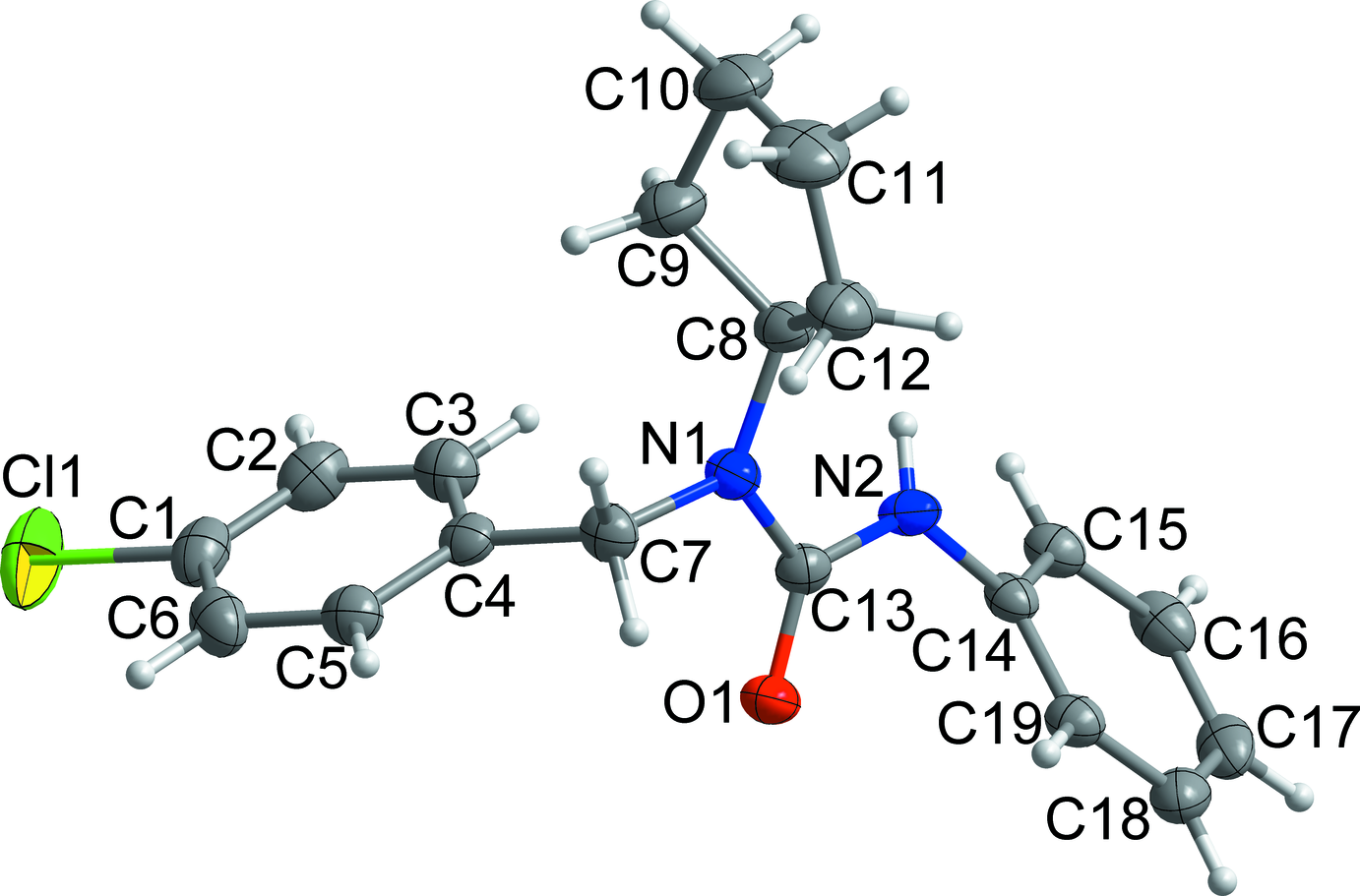

Supplement: Supplementary file 4 [file e-71-0o532-fig1.tif]

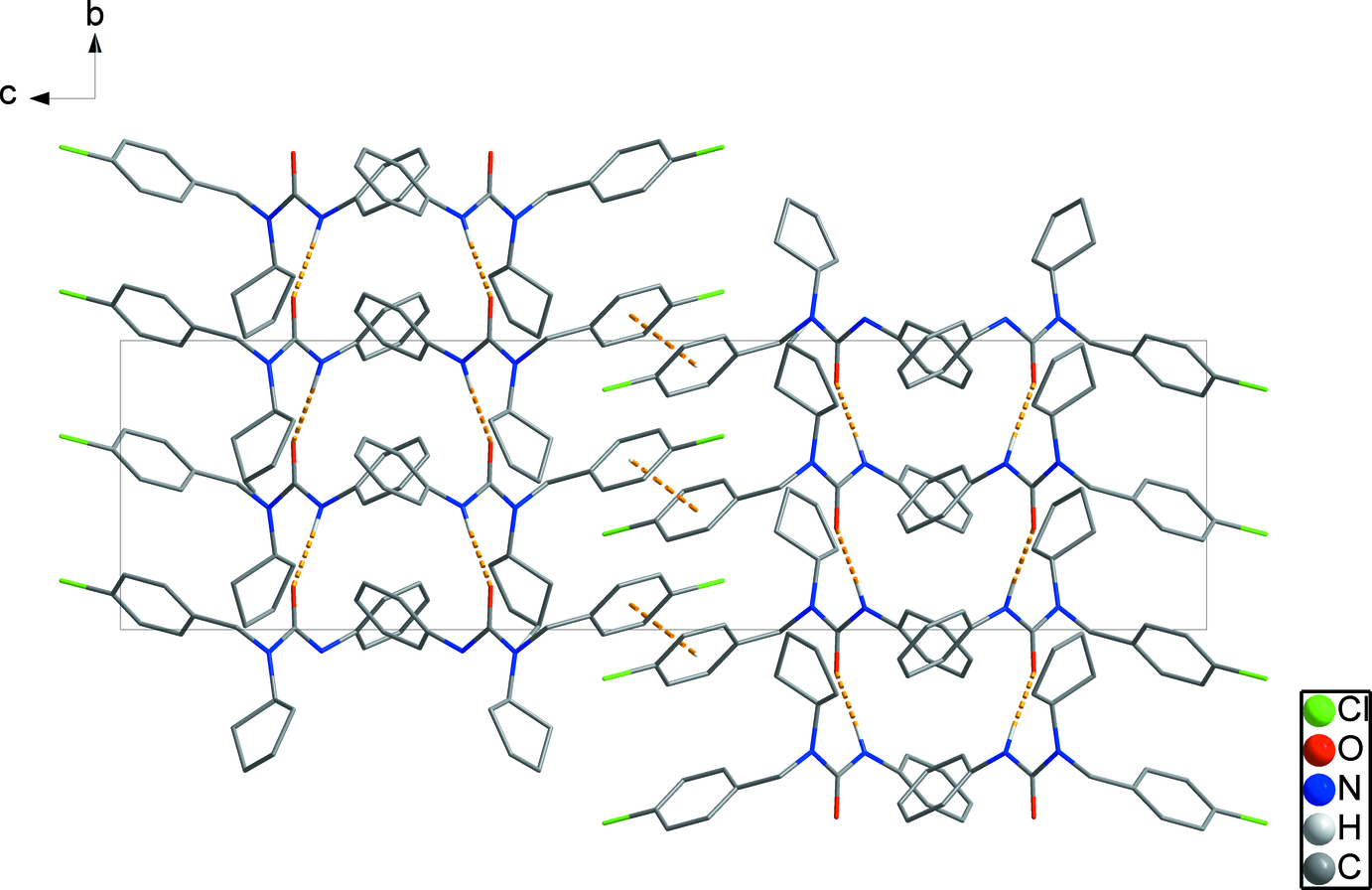

Supplement: Supplementary file 5 [file e-71-0o532-fig2.tif]
